# Relationship between Gepotidacin Exposure and Prevention of On-Therapy Resistance Amplification in a Neisseria gonorrhoeae Hollow-Fiber *In Vitro* Infection Model

**DOI:** 10.1128/AAC.00521-20

**Published:** 2020-09-21

**Authors:** Brian D. VanScoy, Nicole E. Scangarella-Oman, Steven Fikes, Sharon Min, Jianzhong Huang, Karen Ingraham, Sujata M. Bhavnani, Haley Conde, Paul G. Ambrose

**Affiliations:** a Institute for Clinical Pharmacodynamics, Inc., Schenectady, New York, USA; b GlaxoSmithKline, Collegeville, Pennsylvania, USA

**Keywords:** *Neisseria gonorrhoeae*, gepotidacin, pharmacokinetics-pharmacodynamics

## Abstract

Multidrug-resistant Neisseria gonorrhoeae has emerged as a threat to global health. The relationship between gepotidacin exposure and prevention of on-therapy amplification of drug-resistant N. gonorrhoeae was examined using a 7-day hollow-fiber *in vitro* infection model. The study design included both inactive (no-treatment and ciprofloxacin) and active (ceftriaxone) control regimens. Study drug concentration-time profiles were simulated in the *in vitro* system for a single oral 0.5 g ciprofloxacin dose, a single intramuscular 0.25 g ceftriaxone dose, and single or two (8 to 12 h apart) oral gepotidacin doses ranging from 0.75 to 12 g. The initial bacterial burden inoculated in the model was 10^6^ CFU/ml. The gepotidacin, ciprofloxacin, and ceftriaxone broth MIC values for the challenge isolate (N. gonorrhoeae GSK #8) were 0.5, 2, and 0.002 mg/liter, respectively. Samples were collected for enumeration of total and drug-resistant bacterial populations and drug concentrations. The no-treatment control reached a bacterial density greater than 10^8^ CFU/ml over 24 h and remained consistent over the 7-day study period. The bacterial density in the model system of the ciprofloxacin regimen matched that of the growth control throughout the study duration, while the ceftriaxone regimen sterilized the model system by the end of day 1. For gepotidacin, a full dose-response relationship was observed. While failure was observed for the 0.75-, 1.5-, and 3-g single-dose regimens, all gepotidacin single- or divided-dose regimens totaling at least 4.5 g prevented resistance amplification and sterilized the model system. These data are useful to provide gepotidacin dose selection support for treating patients with gonorrhea infections.

## Abstract (precis)

Multidrug-resistant Neisseria gonorrhoeae has emerged as a threat to global health. The relationship between gepotidacin exposure and prevention of on-therapy amplification of drug-resistant N. gonorrhoeae was examined using a 7-day hollow-fiber *in vitro* infection model. The study design included both inactive (no-treatment and ciprofloxacin) and active (ceftriaxone) control regimens. Study drug concentration-time profiles were simulated in the *in vitro* system for a single oral 0.

## INTRODUCTION

Gonorrhea is a common and often painful sexually transmitted disease caused by the bacterium, Neisseria gonorrhoeae. Gonorrhea is increasing in prevalence across many regions of the world, including America, Europe, and Asia (1–4). Worldwide, the World Health Organization estimates 78 million new cases of gonorrhea are acquired each year (5).

A major challenge in treating patients with gonorrhea is the emergence of multidrug-resistant Neisseria gonorrhoeae (6). In a recent study, only 4.9% of isolates were fully drug susceptible when tested against azithromycin, ceftriaxone, ciprofloxacin, gentamicin, penicillin, and tetracycline (7). Alarmingly, 37.8% of these isolates were resistant to at least three of the tested antibiotics. There was great variability among antibiotic *in vitro* susceptibility rates, from 13.1% for penicillin and approaching 100% for ceftriaxone and azithromycin. Interestingly, these data suggest that decreased susceptibility is more frequent for agents with short rather than long half-lives. The contribution of agents with short half-lives to the development of resistance may be the result of the use of a single-dose therapy strategy (8) for such agents when treating patients with gonorrhea.

Gepotidacin (GSK2140944) is a novel bacterial type II topoisomerase inhibitor that dually targets bacterial DNA gyrase and topoisomerase IV by a different mechanism from that of the marketed fluoroquinolone antibiotics (9). Gepotidacin has *in vitro* activity against a wide array of bacterial species, including N. gonorrhoeae (10). To support the selection of dosing regimens for the treatment of patients with uncomplicated urogenital infection caused by N. gonorrhoeae enrolled in a pivotal phase 3 clinical study that is under way (ClinicalTrials.gov identifier NCT04010539), we conducted studies using a hollow-fiber *in vitro* infection model. The objective of these studies was to identify single- and multiple-dose gepotidacin exposures that would prevent the development of on-therapy resistance.

## RESULTS

### *In vitro* susceptibility testing.

The MIC of each study drug against the five clinical N. gonorrhoeae isolates is presented in Table 1. The wild-type (WT) N. gonorrhoeae ATCC 49226 reference strain MIC value was within the expected range for all three challenge compounds (11). Each of the five isolates was susceptible to ceftriaxone (MIC ≤ 0.25 mg/liter) and resistant to ciprofloxacin (MIC ≥ 1 mg/liter) when tested by agar dilution using gonococcal agar (12). The gepotidacin MIC values ranged from 0.25 to 1 mg/liter across the five isolates. MIC values across study drugs were similar when tested by agar dilution using gonococcal agar or by broth microdilution using fastidious liquid growth medium.

**TABLE 1:** Susceptibility testing results and known quinolone resistance mechanisms for the five N. gonorrhoeae clinical isolates and one ATCC reference strain against gepotidacin, ciprofloxacin, and ceftriaxone using broth and agar dilution methods

N. gonorrhoeae isolate	Known resistance mechanism	Broth microdilution MIC using fastidious broth (mg/liter)			Agar dilution MIC using gonococcal agar (mg/liter)		
		Gepotidacin	Ciprofloxacin	Ceftriaxone	Gepotidacin	Ciprofloxacin	Ceftriaxone
ATCC 49226	WT^*a*^	0.5	0.004	0.008	0.25	0.004	0.015
GSK #2	GyrA (S91F, D95A) and ParC (D86N)	0.25	2	0.004	0.5	2	0.004
GSK #4	GyrA (S91F D95G) and ParC (D86N)	1	4	0.03	1	4	0.06
GSK #5	GyrA (S91F D95G) and ParC (D86N)	1	4	0.008	1	4	0.015
GSK #7	GyrA (S91F D95A) and ParC (D86N)	1	4	0.004	1	8	0.008
GSK #8	GyrA (S91F D95A) and ParC (D86N)	0.5	2	0.002	1	2	0.002

a WT, wild type.

### Resistance frequency assay.

The frequency of resistance to gepotidacin assay results are presented in Table 2. The frequency of resistance to gepotidacin at 4× MIC was generally low (<1.5 × 10^−8^), and there were no resistant isolates recovered for three out of the five isolates examined (GSK #2, #5, and #7). For the remaining two isolates (GSK #4 and #8), frequencies of resistance ranged from 8.0 × 10^−8^ to 9.1 × 10^−5^ at 2.5× the MIC value, and gepotidacin resistance was virtually undetectable at 4× the MIC value. The gepotidacin MIC values for the two isolates collected from the gepotidacin-containing agar plates were each determined to be 16 mg/liter, regardless of the gepotidacin concentration of the agar plate.

**TABLE 2:** Resistance frequency for the five N. gonorrhoeae clinical isolates grown on plates containing gepotidacin concentrations at 2.5× and 4× MIC

N. gonorrhoeae isolate	2.5× MIC			4× MIC		
	Replicate no. 1	Replicate no. 2	Replicate no. 3	Replicate no. 1	Replicate no. 2	Replicate no. 3
GSK #2	<5.1 × 10^−10^	<2.6 × 10^−9^	No growth	<5.1 × 10^−10^	<2.6 × 10^−9^	No growth
GSK #4	8.0 × 10^−6^	9.1 × 10^−5^	6.0 × 10^−6^	<1.1 × 10^−8^	<3.1 × 10^−7^	<7.0 × 10^−9^
GSK #5	<1.5 × 10^−8^	<2.6 × 10^−9^	No growth	<1.5 × 10^−8^	<2.6 × 10^−9^	No growth
GSK #7	<9.5 × 10^−9^	<1.1 × 10^−8^	<1.2 × 10^−9^	<9.5 × 10^−9^	<1.1 × 10^−8^	<1.2 × 10^−9^
GSK #8	2.1 × 10^−7^	5.1 × 10^−7^	8.0 × 10^−8^	<1.7 × 10^−9^	<6.5 × 10^−9^	8.7 × 10^−10^

### Pharmacokinetics.

**FIG 1:**
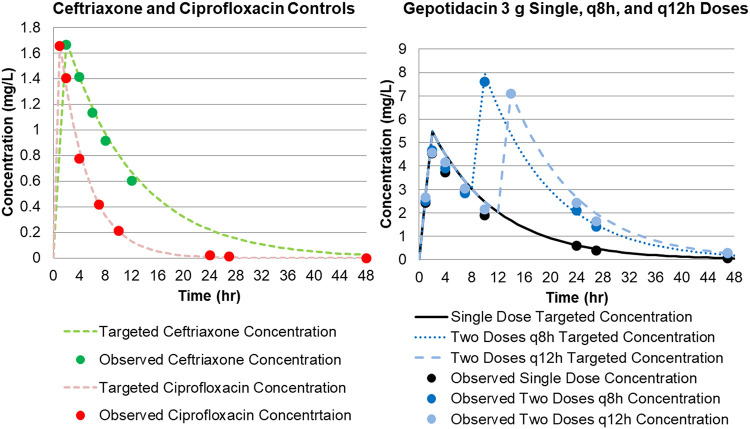
Targeted concentration time profile for gepotidacin, ceftriaxone, and ciprofloxacin in the hollow-fiber *in vitro* infection model, with observed data overlaid.

The agreement between observed and targeted study drug concentrations was high (gepotidacin: coefficient of determination [*r*^2^] = 0.9733, slope = 0.9938, and intercept = 0.0153; ceftriaxone: *r*^2^ = 0.9732, slope = 1.0406, and intercept = 0.0159; ciprofloxacin: *r*^2^ = 0.9536, slope = 0.9316, and intercept = 0.0074), with *r*^2^ indicating goodness of fit and slope representing deviations of 1 ranging from 0.7 to 6.9% percent for the three challenge compounds. Figure 1A and B show the targeted concentration time profile for gepotidacin, ceftriaxone, and ciprofloxacin in the hollow-fiber *in vitro* infection model, with observed data overlaid. These data indicated that each study drug was well simulated in the hollow-fiber *in vitro* infection model.

### Hollow-fiber *in vitro* infection model.

Based on the results of the above-described resistance frequency studies, N. gonorrhoeae GSK #8 met the criteria described in the Materials and Methods, and the results of hollow-fiber *in vitro* infection model studies based on data for this isolate are described below.

**FIG 2:**
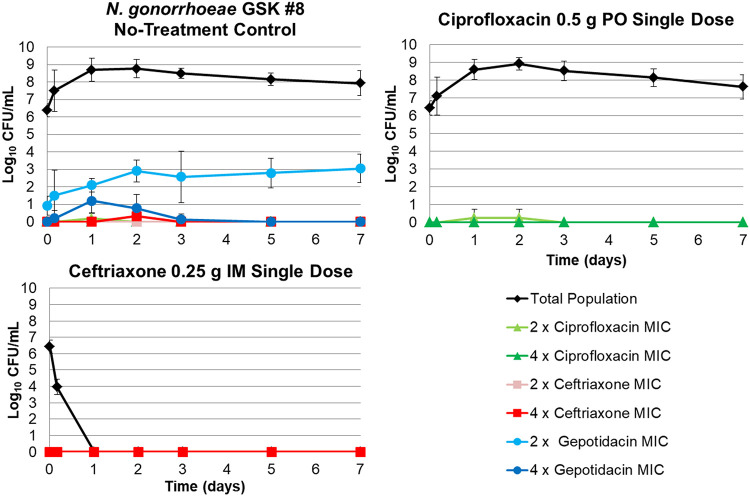
Total and antibiotic-resistant bacterial subpopulations over time for the no-treatment, ceftriaxone 0.25 g i.m., and ciprofloxacin 0.5 g p.o. control dosing regimens based on data for N. gonorrhoeae GSK #8.

The changes in the total and antibiotic-resistant bacterial subpopulations over time for the no-treatment, ceftriaxone 0.25 g intramuscular (i.m.), and ciprofloxacin 0.5 g oral (p.o.) control regimens based on data for N. gonorrhoeae GSK #8 are presented in Fig. 2. The no-treatment control regimen grew well, achieving a bacterial density that approached 9 log_10_ CFU/ml by day 1 and maintaining a generally consistent ratio of the total population to that of the drug-resistant subpopulations over the 7-day study period. Note that the ceftriaxone and ciprofloxacin control regimens performed as expected given the baseline MIC values for N. gonorrhoeae GSK #8. The ciprofloxacin regimen failed (as evidenced by bacterial burdens greater than the initial inoculum at the end of the study duration), while success (i.e., reduction in CFU burden to undetectable levels) was observed for the ceftriaxone regimen.

**FIG 3:**
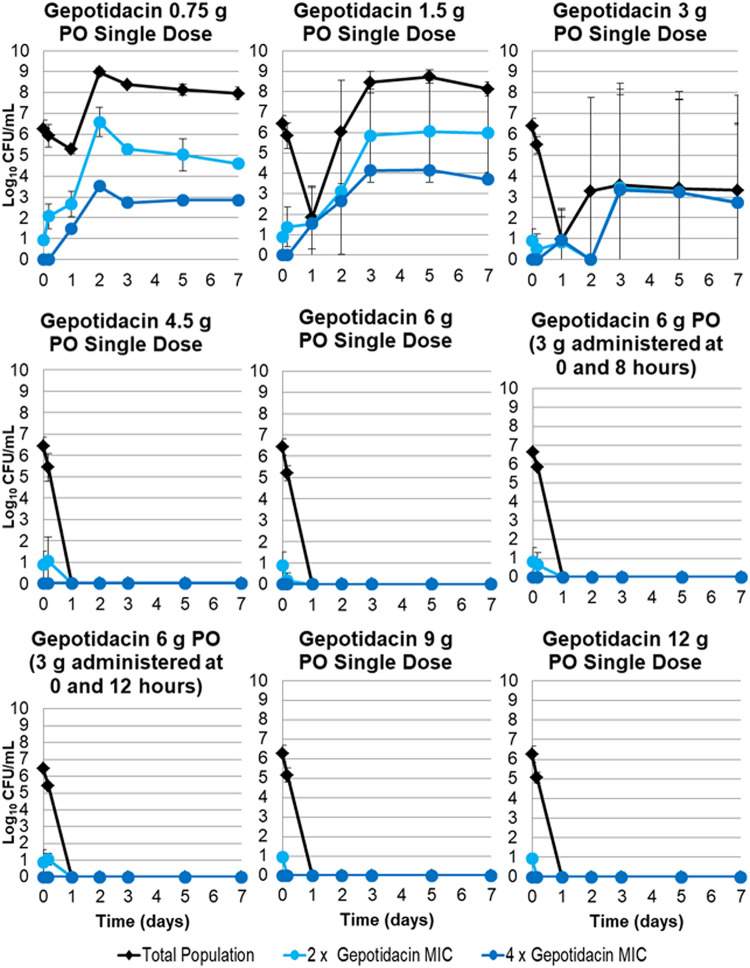
Total population and antibiotic-resistant bacterial subpopulations over time for gepotidacin dosing regimens of 0.75 to 12 g p.o., representing area under the concentration-time curve (AUC) values of 16.2 to 278 mg · h/liter, simulated in the hollow-fiber *in vitro* infection model based on data for N. gonorrhoeae GSK #8.

**FIG 4:**
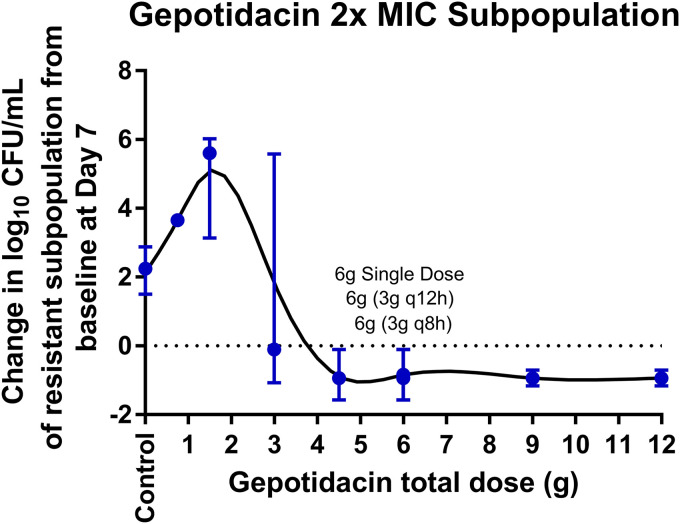
Relationship between gepotidacin total dose and median (min, max) change in log_10_ CFU/ml from baseline of the gepotidacin 2× MIC subpopulation for N. gonorrhoeae GSK #8 on day 7.

The changes in the total population and the drug-resistant bacterial subpopulations over time for the gepotidacin regimens based on data for N. gonorrhoeae GSK #8 are presented in Fig. 3. A full gepotidacin exposure-response relationship was observed over the range of doses from 0.75 to 12 g p.o., which represented area under the concentration-time curve (AUC) values of 16.2 to 278 mg · h/liter. That is, low gepotidacin exposures resulted in regimen failure with drug resistance amplification, and high exposures resulted in the sterilization of the infection model. All gepotidacin single-dose regimens greater than or equal to 4.5 g prevented resistance amplification and sterilized the infection model over the 7-day period. The 6-g gepotidacin dose, split into two equal doses administered at 0 and 8 or 12 h, prevented resistance amplification and sterilized the infection model over the 7-day period. As shown in Fig. 4, the relationship between gepotidacin total dose and the change in bacterial density from baseline of the gepotidacin-resistant subpopulation on day 7 for N. gonorrhoeae GSK #8, isolated from plates containing gepotidacin concentrations at 2× MIC, took the form of an inverted U-shaped curve. The gepotidacin agar MIC values of isolates collected from the 2× MIC gepotidacin-containing plates ranged from 2 to 16 mg/liter. A subset of these isolates tested against gepotidacin in the presence of a broad-spectrum efflux pump inhibitor (Phe-Arg β-naphthylamide dihydrochloride) demonstrated a ≤2-fold decrease in MIC except for one isolate, which had an 8-fold decrease in MIC.

### Genotype characterization.

A total of 51 N. gonorrhoeae isolates with gepotidacin agar MIC values ranging from 1 to 16 mg/liter were recovered on gepotidacin-containing 4× MIC plates from the hollow-fiber *in vitro* infection model studies conducted using isolate GSK #8 (baseline gepotidacin agar MIC, 1 mg/liter). Aside from preexisting substitutions that were also present in the parent isolate (GyrA S91F, D95A; ParC D86N), an additional GyrA A75S or A92T/V substitution was identified in all but two gepotidacin isolates recovered from the 1.5- and 3-g gepotidacin treatment groups. The gepotidacin MIC was 16 mg/liter (16-fold increase in MIC compared to that of the parent isolate) for all except one isolate (MIC 2 mg/liter). Isolates recovered from the 0.75-g gepotidacin treatment group had a 4-fold increase in gepotidacin MIC (MIC, 4 mg/liter) compared to the parent isolate and were found to have a V36I substitution in GyrA.

## DISCUSSION

The overarching goal of the studies described herein was to evaluate gepotidacin dosing regimens to support the dose selection for the pivotal phase 3 clinical study. Specifically, we conducted hollow-fiber *in vitro* infection model studies to identify single- and multiple-dose gepotidacin exposures that would prevent the development of on-therapy resistance. Moreover, we sought to characterize gepotidacin-resistant isolates collected from drug-containing plates.

The relationship between gepotidacin exposure and drug resistance amplification based on data from the hollow-fiber *in vitro* infection took the form of an inverted U-shaped curve. More specifically, low single gepotidacin doses modestly amplified gepotidacin resistance and intermediate doses maximally amplified gepotidacin-resistant subpopulations, while higher doses prevented the amplification of gepotidacin-resistant subpopulations (Fig. 4). Single gepotidacin doses of 4.5 g or more prevented the amplification of resistant subpopulations and sterilized the system by day 7. Two 3-g gepotidacin doses administered at 0 and 8 or 12 h apart also prevented amplification of gepotidacin-resistant subpopulations and sterilized the system by day 7.

Of the gepotidacin-resistant isolates recovered from the hollow-fiber *in vitro* infection model studies, a limited number of substitutions in GyrA (V36I, A75S, and A92T/V) were identified. These results are consistent with both previously conducted *in vitro* resistance frequency assays and also with clinical study results, where GyrA A92T (with a gepotidacin agar MIC increase of ≥16-fold) was the only additional target-based mutation identified in the *in vitro* assay or in isolates recovered posttherapy from urogenital specimens for two subjects (13, 14). In this study, it is likely that the additional GyrA substitutions (V36I, A75S, and A92T/V) contributed to the reduced gepotidacin susceptibility of these isolates, as the gepotidacin MIC values were unaffected by the addition of a broad-spectrum efflux pump inhibitor.

There are a number of strengths of the dynamic hollow-fiber *in vitro* infection model studies described herein relative to *in vitro* resistance frequency assays or other static pharmacokinetic-pharmacodynamic (PK-PD) infection models, including the extended study duration of 7 days. Although N. gonorrhoeae bacterial load can vary significantly for different patient body sites, the bacterial density achieved in this study was similar to larger bacterial burdens as described in the literature (15–18). Additionally, the expected performance of the inactive and active control regimens was easily demonstrated. That is, the no-treatment control regimen grew well, the ciprofloxacin regimen failed, and the ceftriaxone regimen succeeded in the context of the challenge isolate that was resistant to ciprofloxacin and susceptible to ceftriaxone (Fig. 2). Importantly, the gepotidacin-resistant isolates recovered from the hollow-fiber *in vitro* infection model studies contained target-site mutations consistent with those identified from two gepotidacin-treated patients with uncomplicated urogenital gonorrhea enrolled in a phase 2 clinical study (13, 14). The variable efficacy/resistance amplification of the 3-g dose in the hollow-fiber infection model studies was also consistent with the results seen for this dose in the phase 2 clinical study (13, 14). Given these concordant findings, data from studies conducted using the hollow-fiber *in vitro* infection model described herein may be particularly useful to forecast effective regimens for the treatment of uncomplicated urogenital gonorrhea.

There are a few limitations to the work described herein. First, only one isolate was evaluated in the hollow-fiber *in vitro* system, and therefore we are unable to evaluate interisolate variability surrounding the exposure associated with resistance prevention. Second, only one initial bacterial inoculum was evaluated, and given the variability in bacterial burden associated with various infection sites, further work will need to be completed in order to explore the effect that the initial bacterial inoculum may have on gepotidacin activity. Third, the PK profiles simulated in the *in vitro* system represented free-drug plasma exposures rather than exposures at other potential effect sites, such as the pharynx, and thus may not be reflective of other effect site exposures.

In conclusion, we conducted hollow-fiber *in vitro* infection model studies to identify single- and multiple-dose gepotidacin exposures that would prevent the development of on-therapy gepotidacin resistance. The gepotidacin-resistant isolates recovered from the hollow-fiber *in vitro* infection model studies contained additional GyrA mutations consistent with those identified from gepotidacin-treated patients enrolled in a clinical study. These data can be used to support the selection of future gepotidacin dosing regimens for the treatment of patients with uncomplicated urogenital gonorrhea that minimize the potential for on-therapy drug resistance amplification.

## MATERIALS AND METHODS

### Bacteria and antimicrobial agents.

Six N. gonorrhoeae isolates were evaluated in the study described herein. One (ATCC 49226) was purchased from the American Type Culture Collection (ATCC, Manassas, VA). The remaining five isolates were clinical isolates, each from a baseline culture obtained from individual gepotidacin-treated patients with uncomplicated urogenital gonorrhea enrolled in a phase 2 dose-ranging study (13). All five isolates contained a preexisting ParC D86N mutation that likely conferred resistance to fluoroquinolones. However, this mutation compromised dual targeting by gepotidacin, as ParC D86 is a critical residue for interacting with gepotidacin. Clinical resistance to gepotidacin likely developed from the subpopulation containing the preexisting ParC D86N mutation (14). Gepotidacin was provided by GlaxoSmithKline, while ciprofloxacin and ceftriaxone were each purchased from Henry-Schein Medical (Mellville, NY).

### *In vitro* susceptibility testing.

MIC testing of each study drug was performed in triplicate by two methods over 2 days. Agar dilution testing using gonococcal agar (Remel San Diego, CA) was performed according to Clinical and Laboratory Standards Institute (CLSI) guidelines (11). MIC values were also determined by broth microdilution testing using fastidious broth (FB) medium (19), as this liquid medium was used in the hollow-fiber *in vitro* infection model studies described below.

### Resistance frequency assay.

Frequency of resistance to gepotidacin was determined for each of the five clinical isolates. A 5-ml sample was collected from a bacterial suspension in mid-log-phase growth for each isolate. Next, 200-μl aliquots were inoculated onto 25 gonococcal agar plates supplemented with gepotidacin concentrations equal to 2.5× and 4× the gepotidacin agar MIC for each isolate. The inoculated agar plates were incubated in the presence of 5% CO_2_ for a 72-h period. Resistance frequency was determined as the ratio of the growth on the drug-containing agar plates (number of resistant isolates) to that of the starting inoculum. For those drug-containing plates with observable bacterial growth, a subset of isolates were taken for agar-based gepotidacin MIC determination and compared to the baseline.

### Hollow-fiber *in vitro* infection model.

The hollow-fiber *in vitro* infection model used in these studies has been descried previously (20, 21). Briefly, the hollow-fiber *in vitro* infection model is a two-compartment *in vitro* PK-PD system. The challenge isolate is maintained in a peripheral compartment, from which the organism cannot escape. With the aid of computer-controlled peristaltic pumps, study drug and fresh bacterial growth medium are circulated through a central compartment into and out of the peripheral compartment. The pump rates can be set such that virtually any drug concentration-time profile can be simulated in the system. Hollow-fiber cartridge sampling ports allow for the determination of drug and bacteria concentrations over time.

### Hollow-fiber *in vitro* infection model studies.

The isolate selection criteria for the hollow-fiber *in vitro* infection model included the following: (i) a gepotidacin agar MIC of <2 mg/liter, (ii) a gepotidacin resistance frequency greater than 1.0 × 10^−9^ CFU/ml but less than 1.0 × 10^−7^ (in order to select an isolate associated with a known resistant subpopulation), (iii) ciprofloxacin resistance and ceftriaxone susceptibility, and (iv) identical gepotidacin MIC values between the agar and broth methodologies. The inocula for the selected challenge isolate were prepared from overnight cultures on chocolate II agar (Becton, Dickinson and Company, Franklin Lakes, NJ) in the presence of 5% CO_2_. Colonies from overnight cultures were grown to the mid-log-phase and suspended in fastidious broth medium, and the bacterial density was confirmed by optical density using a previously derived bacterial growth curve. Finally, a bacterial suspension volume containing 1 × 10^6^ CFU/ml was inoculated into the peripheral compartment of each hollow-fiber cartridge (FiberCell Systems, Frederick, Maryland) in order to simulate a clinically relevant bacterial burden as described based on the literature (15–18).

Three different study antibiotics were evaluated in the hollow-fiber *in vitro* infection models, ceftriaxone, ciprofloxacin, and gepotidacin. For all study antibiotics, free-drug concentration-time profiles were simulated in the hollow-fiber *in vitro* infection model. Ceftriaxone was infused into the system in a manner that mimicked the concentration-time profile in adult humans following a single 0.25-g intramuscular injection (22) and which represented a component of the current Centers for Disease Control (CDC)-recommended treatment regimen (23). Ciprofloxacin was infused in the system in a manner that simulated the concentration-time profile in adult humans after a single 0.5-g p.o. dose (24) and which represented the former CDC-recommended regimen (25). Gepotidacin was administered in a manner that mimicked adult human concentration-time profiles following a single p.o. dose (26). In order to fully characterize the exposure-response profile for gepotidacin, single p.o. doses ranged from 0.75 to 12 g (26). In addition, a 6-g gepotidacin daily dose was evaluated as two equally divided doses administered either 8 or 12 h apart. All hollow-fiber *in vitro* infection model studies were conducted at least in duplicate and over a 7-day period.

Samples (1 ml) to determine drug bacterial density were collected from the hollow-fiber *in vitro* infection model immediately prior to study antibiotic administration, 4 h post-treatment initiation and on study days 1, 2, 3, 5, and 7. To determine the effect of study antibiotic treatment on the total bacterial population, samples were washed twice using sterile normal saline, serially diluted, and quantitatively cultured on drug-free chocolate agar plates (Becton Dickson, Heidelberg, Germany). To enumerate the antibiotic-resistant subpopulation density, a portion of each bacterial sample was plated on chocolate agar supplemented with each study antibiotic at a concentration equal to 2× or 4× the baseline MIC. Finally, to investigate the effect of efflux, MIC values were determined with and without the presence of 40 mg/liter Phe-Arg β-naphthylamide dihydrochloride (Sigma-Aldrich) for each study day sampled.

Samples for drug concentration determination were collected from the hollow-fiber *in vitro* infection model at 1, 2, 4, 7, 10, 24, 27, 30, and 47 h after treatment initiation. Samples were immediately frozen at −80°C until assayed for drug concentration via liquid chromatography-tandem mass spectrometry.

### Genotype characterization.

PCR amplification was performed on a subset of isolates collected from the resistance frequency and hollow-fiber *in vitro* infection model. The quinolone resistance-determining regions (QRDR) which are “hot spots” for substitutions were analyzed. PCR products were separated, visualized, and sized by electrophoresis on a 1% agarose gel containing ethidium bromide, purified following manufacturer’s instructions using the PCR purification kit, and resuspended in 30 μl sterile distilled H_2_O. PCR products were sequenced by the Sanger method using a BigDye Terminator v3.1 cycle sequencing kit followed by analysis using a 3730*xl* DNA Analyzer; all equipment was from Applied Biosystems (Foster City, California). Sequence alignments were carried out using Lasergene SeqMan software. The gene sequences from reference strain FA1090 as well as from the parent strain were aligned with the mutant gene sequences to determine nucleotide differences.

### Drug assay.

Samples were assayed by liquid chromatography-tandem mass spectrometry (LC-MS/MS; Sciex 5500, Framingham, MA). The standard curves for ciprofloxacin, ceftriaxone, and gepotidacin were linear, with *r*^2^ values of 0.99 for all compounds over ranges from 0.01 to 2.00, 0.25 to 50, and 0.01 to 25 mg/liter, respectively. All standard curves and quality controls were prepared in study matrix (FB) and processed concurrently with collected samples. For ciprofloxacin, the interassay percent coefficient of variation (%CV) for the quality control samples at concentrations of 0.030, 0.350, and 1.40 mg/liter were 7.41%, 3.95%, and 3.17%, respectively. For ceftriaxone, interassay %CV for the quality control samples at concentrations of 0.750, 8.75, and 35.0 mg/liter were 10.2%, 4.86%, and 4.22%, respectively. The lower limit of quantification was 0.01 mg/liter for ciprofloxacin and gepotidacin and 0.25 mg/liter for ceftriaxone.
